# Synchrotron Radiation X-Ray Microfluorescence Reveals Polarized Distribution of Atomic Elements during Differentiation of Pluripotent Stem Cells

**DOI:** 10.1371/journal.pone.0029244

**Published:** 2011-12-16

**Authors:** Simone C. Cardoso, Mariana P. Stelling, Bruna S. Paulsen, Stevens K. Rehen

**Affiliations:** 1 Instituto de Física, Universidade Federal do Rio de Janeiro, Rio de Janeiro, Brazil; 2 Instituto de Ciências Biomédicas, Universidade Federal do Rio de Janeiro, Rio de Janeiro, Brazil; 3 Hospital Universitário Clementino Fraga Filho, Universidade Federal do Rio de Janeiro, Rio de Janeiro, Brazil; Cuban Neuroscience Center, Cuba

## Abstract

The mechanisms underlying pluripotency and differentiation in embryonic and reprogrammed stem cells are unclear. In this work, we characterized the pluripotent state towards neural differentiated state through analysis of trace elements distribution using the Synchrotron Radiation X-ray Fluorescence Spectroscopy. Naive and neural-stimulated embryoid bodies (EB) derived from embryonic and induced pluripotent stem (ES and iPS) cells were irradiated with a spatial resolution of 20 µm to make elemental maps and qualitative chemical analyses. Results show that these embryo-like aggregates exhibit self-organization at the atomic level. Metallic elements content rises and consistent elemental polarization pattern of P and S in both mouse and human pluripotent stem cells were observed, indicating that neural differentiation and elemental polarization are strongly correlated.

## Introduction

The hallmark of embryonic stem (ES) cells is their pluripotency [1], which can also be achieved by enforced expression of specific transcription factors in somatic cells [2], [3]. ES and induced pluripotent stem (iPS) cells, which can be referred to as pluripotent stem cells, have the ability to initiate differentiation by aggregating as embryo-like structures called embryoid bodies (EBs) [4], [5]. Pluripotent stem cells growing as EBs go through a dynamic differentiation process, starting with the formation of a primitive endoderm layer on the surface, followed by the development of cystic cavities and the arising of cell phenotypes of the three somatic germ layers [6], [7]. These dynamics shows that EBs are able to mimic the embryonic development to a certain degree [8]. Furthermore, EBs can also be directed towards a specific differentiation pathway by the addition of inducers such as retinoic acid [9], which increases the percentage of cells committed towards the neural phenotype.

In the past ten years, many scientists have spent a great effort in order to develop non-destructive techniques for investigating biological structures on a micrometric or sub-micrometric scale [10]. Synchrotron radiation X-ray fluorescence spectroscopy (SR-XRF) fulfills most of the criteria for such analysis. It is a non-destructive, multi-elemental analysis technique [11] that does not require any complex pretreatment of samples [12]. Its microprobe has better elemental sensitivity - in the range of parts per million - than most charged particle probes. Moreover, it allows imaging and elemental mapping of samples in the micrometer scale, allowing chemical determinations of heterogeneous samples.

SR-XRF has been mainly used for total elemental mapping analyses [13], [14] and localization of specific elements within a sample [15], [16], [17], [18]. Post mortem human brains are an example in which SR-XRF was used to compare the impact of disease on metallic elements distribution within the central nervous system [19].

Analysis of pluripotent stem cell neural differentiation has never been carried out at the atomic level. Here, we explored SR-XRF to describe the behavior of atomic elements in pluripotent stem cells undergoing differentiation as a model to study neural development.

Our results show that naive EBs derived from ES and iPS cells present a consistent pattern of elemental distribution which is unique for both species, mouse and human. Nevertheless, after neural stimulation, both murine and human cells raise the content of metallic elements copper and zinc and present the same elemental pattern: phosphorus and sulfur polarize during neural differentiation, while copper and zinc are widely spread along the EBs. These data will be useful for future studies of disease models, such as patient-derived iPS cells compared to healthy subjects-derived iPS cells undergoing differentiation *in vitro*.

## Materials and Methods

### ES and iPS cell culture

R1 mouse embryonic stem (ES) cells [20] and mouse induced pluripotent stem (iPS) cells [9] were maintained in a mixture of high-glucose Dulbecco's modified Eagle medium (DMEM) and F12 (1∶1) supplemented with 15% Knockout™ Serum Replacement (KSR), 100 mM glutamine, 55 mM 2-mercaptoethanol, 100 µM non-essential amino acids (all from Gibco Invitrogen Corporation, USA) on 2% gelatin-coated dishes covered with mitomycin C-treated (10 µg/ml; Sigma) mouse embryonic fibroblasts (MEF). As a source of leukemia inhibitory factor (LIF), we used conditioned medium (1∶500 dilution) from CHO cell cultures that had been transduced with a LIF-encoding vector. Cells were passaged every three days.

H9 human ES cells [21] were cultured in a mixture of high-glucose DMEM and F12 (1∶1) supplemented with 20% KSR, 200 mM glutamine, 55 mM 2-mercaptoethanol, 100 µM non-essential amino acids and 8 ng/mL FGF-2 (also from Gibco Invitrogen Corporation, USA) on 2% gelatin-coated dishes covered with mitomycin C-treated MEF cells. H9 (ES cells) were passaged every five days. All cells were maintained at 37°C in humidified air with 5% CO_2_.

### Embryoid body formation and neural induction

#### Formation of embryoid bodies with mouse pluripotent stem cells

Confluent mouse pluripotent stem cells were transferred to a gelatin-coated substrate for 24 hours and fed with the same medium as on the feeder layer. The cells were then dissociated to a single-cell suspension by enzymatic treatment with TrypLE™ Express (Invitrogen) at a concentration of 2x10^5^ cells/mL and 40 µL hanging drops were plated on 100 mm non-adherent petri dishes covers (Corning). The medium used from this point was similar to mouse ES medium except for the use of fetal bovine serum instead of KSR and the absence of LIF. After 48h, the aggregates were collected into 60 mm non-adherent petri dishes in 7 mL of medium per plate. Embryoid bodies (EBs) cultivated in this condition until day 4, were named naive EBs. At this point this group was fixated with 4% paraformaldehyde for analysis and the remaining EBs were kept in culture for neural induction. Retinoic acid (RA - Sigma-Aldrich Corp., St. Louis, USA) was used at 5 µM as a neural inducer for 4 days in group 2–‘8 days neuro EBs’. Group 3 was cultivated for the same time with DMSO, the RA vehicle, as a control–‘8 days control EBs’.

#### Formation of embryoid bodies with human embryonic stem cells

Confluent H9 cells were dissociated to a single-cell suspension, by enzymatic treatment with TrypLE Express (Invitrogen), at a concentration of 2×10^5^ cells/mL and 40 µL hanging drops were plated on 100 mm non-adherent petri dishes covers. The medium used from this point was similar to hES medium except for the use of 15% instead of 20% KSR and the absence of FGF-2. After 48 h, the aggregates were collected to 60 mm non-adherent petri dishes in 7 mL of medium per plate. EBs were cultivated in this condition until day 7, these were considered naive EBs. At this point, one group was fixated for analysis and the remaining EBs were kept in culture for neural induction, this second group was transferred to neural induction medium–‘14 days neuro EBs’ - and a third group was kept in regular human EB medium as a control of spontaneous differentiation–‘14 days control EBs’. Neural induction medium consisted of a mixture of high-glucose DMEM and F12 (1∶1), 1 µg/mL bovine heparin, 1% non-essential amino acids, 1% N2 supplement and 0.5 ng/mL FGF-2 (all, but heparin, from Gibco Invitrogen Corporation, USA). Groups 2 and 3 were cultivated in these conditions for 7 days, enclosing a total of 14 days of differentiation.

#### Formation of neurospheres with human embryonic stem cells

H9 pluripotent colonies cultured on matrigel (BD Biosciences) with mTeSR medium (Stem Cell Technologies) were differentiated into neurospheres according to Baharvand *et al* [22]. Briefly, cells were cultured for 18 days in the following differentiation medium: mixture of high-glucose DMEM and F12 (1∶1), 5 % KSR, 200 mM glutamine, 55 mM 2-mercaptoethanol and 100 µM non-essential amino acids. For the first 6 days, 4 µM retinoic acid was added to the medium. From 6^th^ to the 12^th^ day cells were cultured in differentiation medium only, until this step medium was changed in alternate days. Finally from the 12^th^ to the 18^th^ day cells were cultured in differentiation medium with the addition of 25 ng/mL FGF-2. At this step medium was changed every day. During this period, cells differentiated and formed neural-tube like structures that, at the end of these 18 days, were dissected from the plate and cultured in the following medium for additional 7 days: neurobasal medium, 1% N2 supplement and 2% B27 supplement.

### Embryoid bodies immunostaining

EBs were collected from plates and immediately fixed in 4% paraformaldehyde, and then the material was processed for obtaining 10 µm cryosections (Figure S1) as previously described [23].

Immunofluorescence staining was performed using the following primary antibodies: anti-Nestin (1∶100 - Chemicon) and anti-beta-III tubulin (1∶100 - Chemicon). DAPI (1.0 µg/mL) was used for nuclei staining. Embryoid bodies immunostaining was performed as follows - 10 µm frozen sections were first taken to a immunogenic recovery in citrate buffer (0.01 M–pH 6.0) boiling bath for 10 minutes. Then sections were permeabilized with 0.5% Triton X-100 (Sigma), blocked in 5% bovine serum albumin (Invitrogen) and incubated with primary and secondary antibodies for 60 and 90 minutes, respectively.

### Cell nuclei density analysis

Embryoid body cell nuclei density was assessed by DAPI nuclei staining on frozen sectioned EBs. All samples were prepared for analysis and images were captured and quantified per group. Images were divided in left and right sides and nuclei side-to-side percentage was determined.

### Embryoid body and neurosphere preparation for XRF analysis

**Figure 1:**
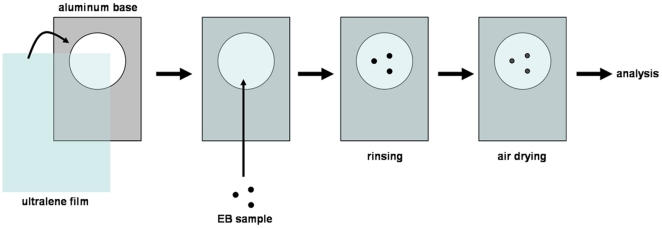
Sample assembly. In order to fixate EB samples on ultralene film, an aluminum base was used and film was attached to it with regular tape. EBs were water rinsed and air dried on this assembled piece.

EBs and neurospheres were collected for fixation in 4% paraformaldehyde (Sigma) for 30 minutes. The cell aggregates were then washed with PBS and kept at 4°C until analysis. Before measurements, samples were carefully placed on ultralene film (transparent to X-Ray), rinsed with distilled water and air-dried for analysis (Figure 1).

### Synchrotron Radiation - X-ray fluorescence

**Figure 2:**
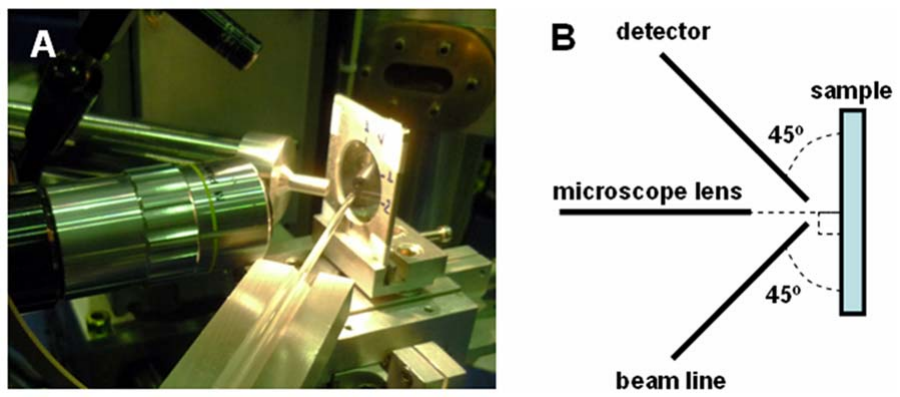
Beamline geometry. A) XRF line set up at the LNLS. B) Beamline geometry (view from the top).

The SR-XRF analyses in this investigation were performed at the X-ray fluorescence beamline of the Brazilian Synchrotron Light Source – LNLS [24], Campinas (SP), Brazil, at room temperature and ambient pressure. A polychromatic (Emax = 22 keV) beam coming from a storage ring was focused by a fine conical capillary capable to achieve 20 µm spatial resolution (Figure 2). The typical measurement area was 200×200 µm^2^ and the measurement time was 150 sec/point.

The fluorescence emitted by trace elements was detected using a Si(Li) detector (energy resolution of 165 eV at 5.9 keV) placed at 90° from the incident beam and close to the sample at a distance of 22 mm.

Controls included scanning of plain ultralene film, paraformaldehyde rinsed film, PBS rinsed film and also an area on the ultralene film that previously contained an EB. All controls showed no significative background signal.

### Spectrum analysis and elemental mapping

All the spectra were analyzed using the Quantitative X-ray Analysis Software (QXAS) package, which is a conventional program for spectrum analysis [25]. Each data point obtained from the analyzed spectra was combined into a numerical matrix used to generate a heat map in order to outline elements distribution and facilitate sample comparison. Map combination was made in PyMca [26] for detection of elemental overlapping areas.

### Left-right intensity maps

In order to quantify and statistically analyze elemental polarization on the atomic maps, each numerical matrix generated from the acquired spectra was divided in two halves, left and right. The highest photon content was always placed to the left in order to orientate matrices and maps in a similar manner. These halves had their photon intensity summed and normalized by the matrix total photon content. Left-right bar graphs were generated and paired-T test was applied to these values (Prisma software).

### Detected radiation per area analysis

Trace elements content of EBs derived from embryonic and induced pluripotent stem cells were related to detected radiation per irradiated area. Briefly, the numerical matrices were analyzed for total elemental content normalized per irradiated area, generating an average value that could be used for comparing EBs of different sizes. Bar graphs were generated and paired-T test was applied to these values (Prisma software).

## Results

### Synchrotron radiation-X ray fluorescence is suitable for embryoid bodies multi-elemental analyses

SR-XRF detection range covers potentially all elements, although optimal detection of K-edge photons occurs between elemental atomic numbers ranging from 14 to 30. Each acquired spectrum contains every element present in the irradiated area within the detection range. These spectra are translated into a heat map that highlights areas containing the highest and lowest elements concentrations, which allows comparisons between a single element in different EBs.

In order to irradiate EB samples, we developed a procedure that consists on the following steps: 1) EB fixation in paraformaldehyde; 2) plated on ultralene covered aluminum molds; 3) scanning; 4) map construction and 5) map analysis.

In order to check sample integrity from step 1 to 2, we recovered EBs for cryosections and further, performed immunostaining analyses. We verified that it was possible to detect the typical ectodermal marker nestin in these samples and that nuclei content was intact.

Scanning procedures (step 3) required embryoid bodies to be exposed to short-term synchrotron radiation. Therefore, we investigated possible damages caused by the high flux of synchrotron radiation in the samples. One first short-term exposure was used to generate a control spectrum, and after a 1,000s exposure, a second spectrum was acquired and both spectra were compared (Figure S2). This analysis revealed that synchrotron radiation does not alter EB atomic structure at the level of XRF sensitivity, making this technique suitable for EB scans.

In this work, we detected the following elements within EBs: phosphorus (P), sulfur (S), chlorine (Cl), potassium (K), calcium (Ca), chromium (Cr), manganese (Mn), iron (Fe), nickel (Ni), copper (Cu) and zinc (Zn). Cl was within detection range but was not considered for this work as this element did not behave in a pattern. Also, although Cr and Mn were in the SR-XRF detection range, due to their very low levels, they were considered ultratrace elements. Therefore, these three elements were excluded from the analyses.

### Neural induction enhances atomic polarization and raises metallic elements in murine embryoid bodies

**Figure 3:**
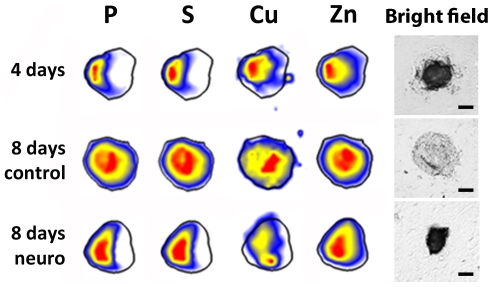
Atomic distribution within murine embryoid bodies. Top line: 4 days old murine embryoid body; Middle line: 8 days old control murine embryoid body; Bottom line: 8 days old neural induced murine embryoid body. Atomic elements are shown on the top line above the heat maps. All derived from the murine embryonic stem cell line R1. Maps are representative of all samples. Bright field images scale bar: 25 µm.

**Figure 4:**
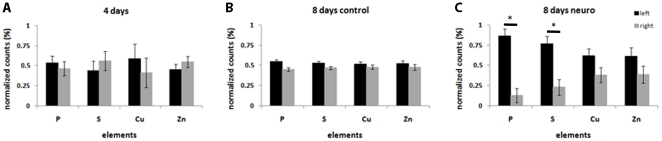
P and S polarization is significant on 8 days old neural induced murine embryoid bodies. Left and right intensity of P, S, Cu and Zn in R1 embryoid bodies. A) 4 days old group; B) 8 days old control group; C) 8 days old neural induced group. Left side is represented by black bars and right side is represented by grey bars, *p<0.05.

Murine EBs were scanned and analyzed at three developmental stages. All, except ultratrace elements, presented a consistent pattern, while P, S, Cu and Zn showed a specific spatial distribution and were carefully analyzed (Figure 3). It can be noticed that elemental distribution is not homogeneous. Left-to-right map analyses (Figure 4) comprising all analyzed EBs (Table 1) showed a slight polarization trend. It is important to emphasize that for left-to-right analyses, all maps were built with the highest element content towards the left side, despite being scanned in random positions. Thus, all maps could be oriented in a similar manner.

**Table 1:** Number of irradiated embryoid bodies per group.

Mouse cells	4 days	8 days control	8 days neuro
R1	n = 6	n = 3	n = 3
MiPS	n = 5	n = 3	n = 3

Human cells	7 days	14 days control	14 days neuro
H9	n = 8	n = 3	n = 5

In order to evaluate if elemental polarization occurs after specific differentiation, EBs were cultured in the presence or absence of retinoic acid (RA) for additional 4 days. In this condition, RA-treated EBs develop a neural-enriched cell population [9]. EBs were analyzed by SR-XRF and heat maps were built (Figure 3 – middle and bottom lines). The elemental maps bring to light a unique polarization pattern. Elemental polarization is observed in mature EBs undergoing neurogenesis induced by RA, while spontaneously differentiated EBs do not present this pattern. Elements, such as P and S, tend to be located on one side of the sphere in EBs cultured for 4 days and are even more polarized in neural induced EBs. Left-to-right maps showed statistical difference between P and S from left to right in neural induced EBs (Figure 4C). While spontaneously differentiated EBs present a spread occurrence of both elements (Figure 4B). On the other hand, Cu and Zn are homogeneously distributed along the sphere in all conditions.

**Figure 5:**
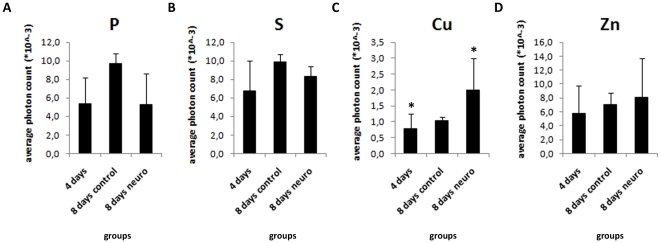
Murine embryoid bodies show similar levels of P, S, Cu and Zn at different stages of differentiation. Measure of elemental content normalized per irradiated area, A) P; B) S; C) Cu; D) Zn, *p<0.05.

P and S contribute to cell metabolism as they are present in nucleic acid and protein composition. As these elements seem to be polarized on neural induced EBs, we investigated whether the content of P and S per area could vary between control and neuro EBs from 4 days to 8 days. We found that P and S content is not altered during EB development (Figure 5A and 5B). Therefore, their polarization consists in the concentration of these elements to specific areas in neuro-induced EBs, rather than rising from total element content.

Neural tissues are enriched in metallic elements such as Cu, Zn and Fe [13]. Despite not being polarized elements, we investigated whether the detected metallic elements Cu and Zn content rise after neural induction. We found that Cu occurs 2.6 times higher on 8 days neuro EBs compared to 4 days EBs. Zn levels are statistically unaltered, however; 8 days neuro EBs present 1.37 times higher Zn content compared to 4 days EBs. These results suggest that metals accumulation may also be associated to neural differentiation process *in vitro*.

**Figure 6:**
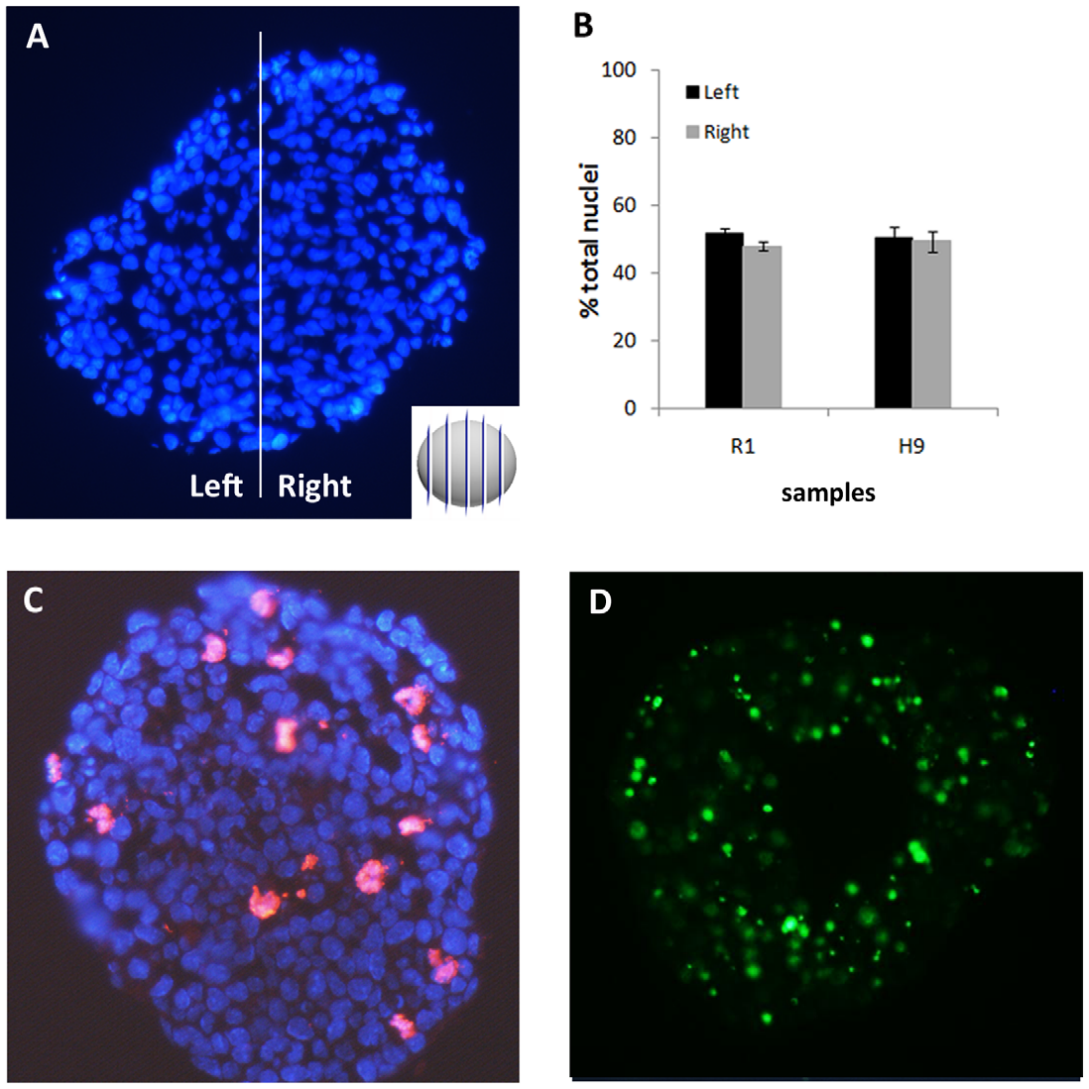
Atomic polarization is independent of cell density, proliferation and death. A) Example of a H9 14 days old neural induced embryoid body divided in half for nuclei quantification; B) Symmetry measure: left and right percentage of nuclei for neural induced R1 (n = 5) and H9 (n = 4) embryoid bodies; C) Example of PH3 staining (red) on a murine embryoid body; D) Example of TUNEL staining (green) on an H9 embryoid body. All DAPI (blue).

We have also investigated whether cell density, proliferation and death could influence elemental distribution by the formation of high cell density areas within EBs. Cell nuclei density was quantified on random slices in order to obtain unbiased records (Figure 6A and 6B). Results show no difference between left and right sides. Also, although EBs from all groups present no morphological differences aside from size (Figure S3), EBs placed on ultralene film present dark areas. These tridimensional regions appear dark due to the contrast method used to photograph the samples. These regions possess tridimensional shape after drying on the ultralene film, nevertheless they are not areas of higher cell density, as cell nuclei distribution in EBs is homogeneous, and elements are not necessarily present in higher concentrations in these regions (Figure S4).

Mitotic figures and dying cells were evenly distributed on EBs (Figure 6C and 6D, respectively). These evidence corroborate the fact that P and S polarization is a hallmark of neural differentiation and is not influenced by EB cell density or its symmetric nature.

All analyses were also performed in murine iPS cells. Results with iPS EBs were similar to EBs derived from ES cells (Figure S5), confirming that P and S polarization occurs both in pluripotent murine cells upon neural stimulation.

In summary, the total elemental spatial pattern was consistently detected in the analyzed murine stem cell lines, but absent in mouse embryonic fibroblasts (data not shown), indicating its association with pluripotency.

### Neurogenesis-associated atomic polarization also occurs in human embryonic stem cells

**Figure 7:**
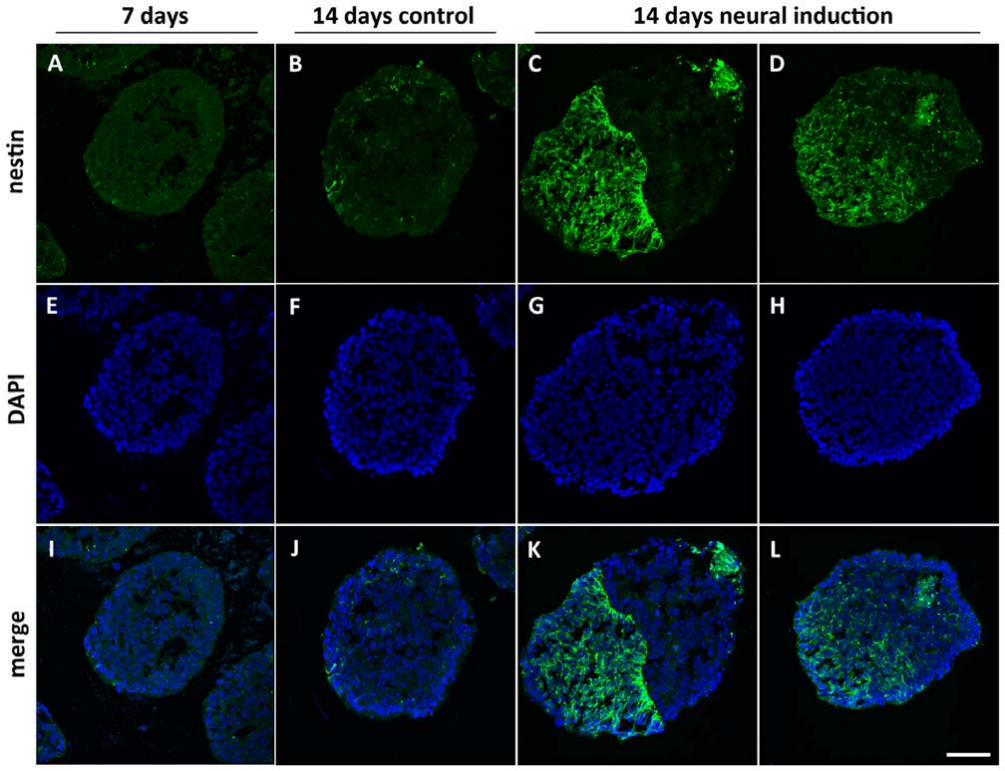
Nestin expression is polarized after neural induction in human embryoid bodies. Nestin staining of H9 embryoid bodies, A) 7 days old group; B) 14 days old control group; C and D) 14 days old neural induced group. E to H) DAPI staining; I to L) merge. The embryoid bodies depicted in this figure are corresponding examples of the total population. Scale bar 50 µm.

**Figure 8:**
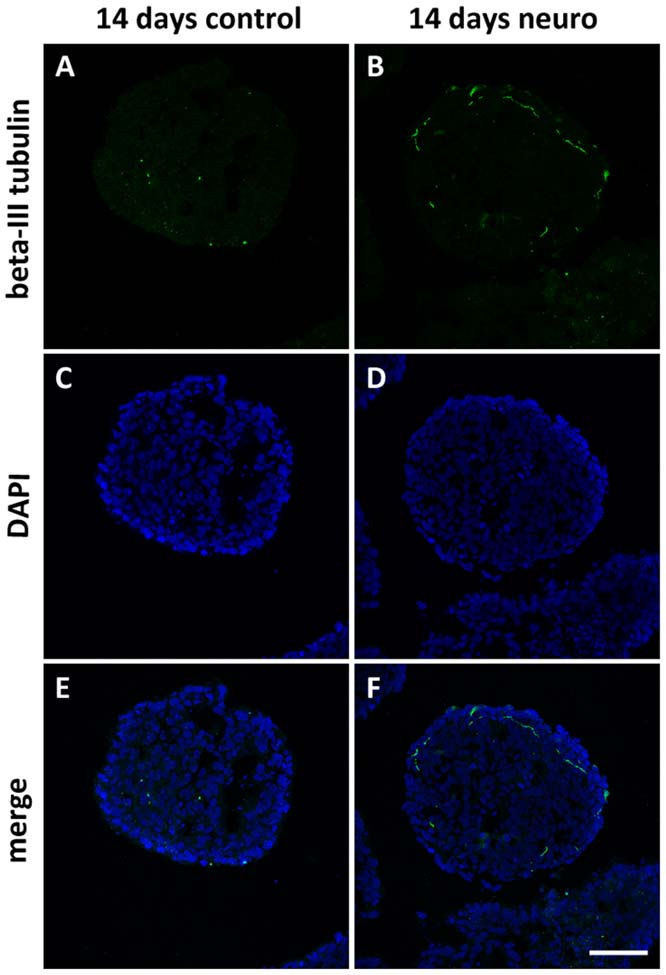
Beta-III tubulin staining is exclusive to 14 days old neural induced embryoid bodies. Beta-III tubulin staining of H9 embryoid bodies (green), A) 14 days old control group; B) 14 days old neural induced group; C and D) DAPI staining (blue); E and F) merge. Scale bar 50 µm.

**Figure 9:**
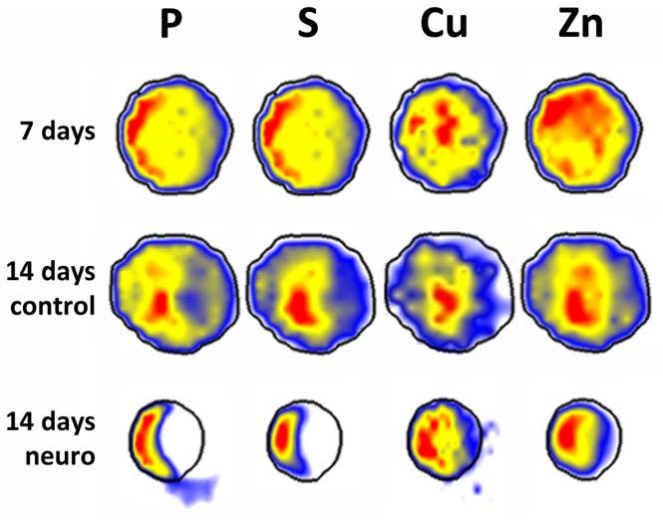
Atomic distribution within human embryoid bodies. Top line: 7 days old H9 embryoid body; Middle line: 14 days old control H9 embryoid body; Bottom line: 14 days old neural induced H9 embryoid body. Elements are shown on the top line above the heat maps. Maps are representative of all samples. Bright field images scale bar: 50 µm.

**Figure 10:**
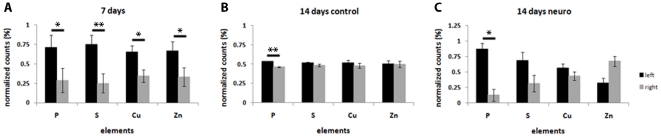
Human pluripotent cells-derived embryoid bodies retain P and S polarization under neural stimulus. Left and right intensity of P, S, Cu and Zn in H9 embryoid bodies. A) 7 days old group; B) 14 days old control group; C) 14 days old neural induced group. Black bars correspond to the left side, grey bars correspond to the right side of atomic maps, *p<0.05 and **p<0.01.

**Figure 11:**
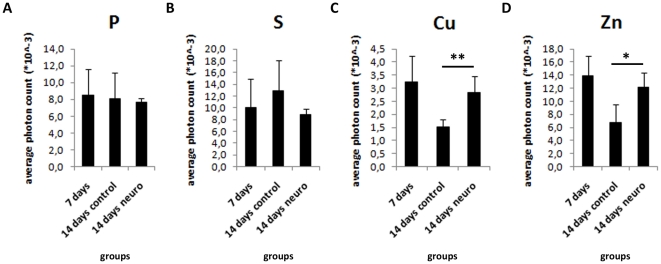
Human embryoid bodies show similar levels of P, S, Cu and Zn at different stages of differentiation. Measure of elemental content normalized per irradiated area, A) P; B) S; C) Cu; D) Zn, *p<0.05; **p<0.01.

Human EBs were analyzed by SR-XRF in order to evaluate if the pattern observed in murine cells is also present in human cells. Detection of markers was performed in parallel to SR-XRF analyses and results showed that the early neural marker nestin (Figure 7) and the neuronal marker beta-III tubulin (Figure 8) are expressed in a polarized manner in neural induced EBs (14 days neuro group). Human ES cells exhibit slower development when compared to mouse ES cells. Indeed, human control EBs, which did not receive neural stimulus, presented a discrete and diffused nestin staining after 7 days of maturation. Therefore, we focused on elemental analyses between 7 and 14 days of development. Figure 9 depicts representative maps of one EB from each group (see Table 1 for the total number of analyzed human EBs). Unlike murine EBs cultured up to day 4, signs of polarization were detected earlier in human EBs cultured up to day 7 (Figure 9 – top line and Figure 10A). Although 14 days control EBs lose this polarization pattern (Figure 9 – middle line and Figure 10B), 14 days neuro EBs keep only P and S polarization (Figure 9 – bottom line and Figure 10C), losing Cu and Zn polarization, resulting in a similar pattern obtained in murine neural stimulated EBs. Cell density analyses were also performed (Figure 6B) showing that H9 EBs have homogeneous mass, despite cavity formation. In addition, analyses of P, S, Cu and Zn content per area were performed (Figure 11) showing that the polarized elements P and S are present in the same amounts in all groups. Also, metallic elements Cu and Zn content rise when comparing 14 days control EBs to 14 days neuro EBs (Figure 11C and D).

**Figure 12:**
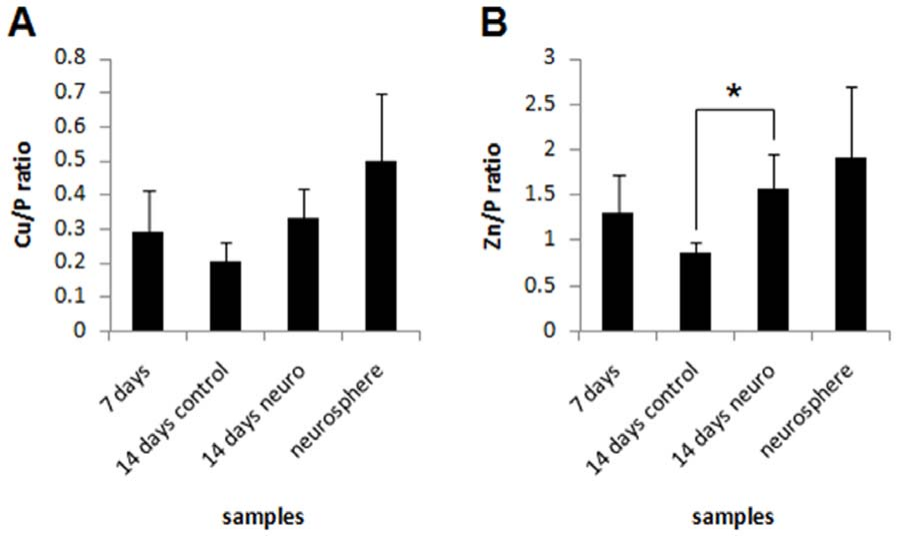
Human embryoid bodies and neurospheres present comparable amounts of metallic elements. Embryoid bodies and neurospheres metals were normalized by phosphorus content. A) Cu/P ratio; B) Zn/P ratio. *p<0.05.

Neurospheres contain a pure population of neural cells that are produced through direct differentiation of ES cells to neural cells, while EBs initially differentiate into cells from the three germ layers, mimicking development, and further differentiate into neural cells if exposed to neural-inducing agents. Comparison between human EBs and neurospheres was also performed in order to analyze metallic elements content between a neural-enriched cell population (EBs) and a pure neural cell population (neurospheres). Results are expressed as normalized Cu and Zn content (Figure 12). Neural induced EBs and neurospheres possess comparable amounts of Cu and Zn. Moreover, both cell aggregates contain slightly higher levels of those metals compared to non-induced 14 days old control EBs.

These data show that murine and human naive EBs present different elemental distribution, but upon neural stimulation they both present P and S polarization and metallic elements accumulation.

### Atomic elements follow a distribution pattern according to the developmental stage and differentiation stimulus

**Figure 13:**
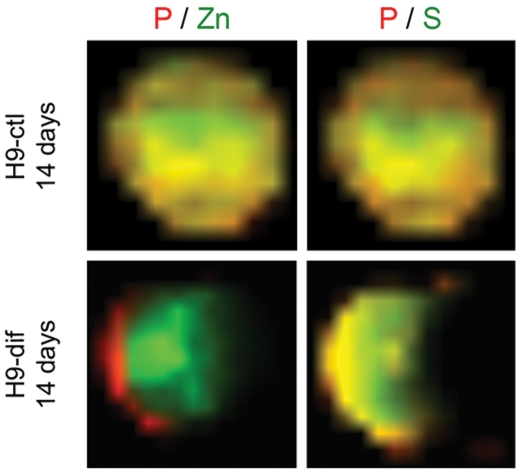
Phosphorus co-localizes with sulfur, but does not overlap with zinc in neural induced EBs.

Figure 13 shows an overlapping map of control and neural induced human EBs. As seen, yellow areas correspond to co-localization. P, S and Zn co-localize in spontaneously differentiated EBs, but when neural induced EBs were analyzed, it was possible to observe that P and S overlap, and do not co-localize with Zn. This same observation is consistently confirmed by the left-right intensity graphs (Figure 10C), whereas Zn tends to polarize to the right side, while P and S polarize to the left.

In summary, results show a consistent elemental P and S polarization pattern, exclusive to areas of higher Cu and Zn concentration in neural induced embryoid bodies. This phenomenon is present in both murine and human pluripotent cells, which is evident after induction of neurogenesis.

## Discussion

Here we demonstrate that effective elemental distribution, content and polarization associated with neurogenesis in murine and human pluripotent stem cells derived embryoid bodies, can be assessed by the use of SR-XRF. There are many analytical techniques that enable determination of trace elements distribution in biological samples, however; most of them use high-vacuum systems, which may alter the cellular material itself and the elements distribution [27]. In addition, they possess spatial resolution limitations. We suggest that SR-XRF is, therefore, one of the best-suited techniques for pluripotent stem cells multi-elemental analysis in the present.

The elements here detected are the following: P, S, K, Ca, Fe, Ni, Cu and Zn. SR-XRF does not distinguish free ions from molecule-attached atoms, and all detected elements presented a consistent pattern, occurring in all samples and in related areas. This fact reinforces that these elements are participating in cell growth and differentiation, being positioned in specific areas. Therefore, we conclude that embryoid bodies exhibit self-organization at the atomic level.

Analyses were performed in mouse and human EBs during early stages of development, whereas a trend for polarization of the elements P, S, Cu and Zn were observed in human samples (Figure 9 and Figure 10). Recently, Berge and colleagues [28] demonstrated a similar polarization pattern in early EBs; however, in that case, the polarized compounds were not atomic elements, but effectors of Wnt signaling involved in axis formation.

In order to investigate if polarized and unpolarized elemental patterns change after maturation, analyses were done in spontaneously differentiated and neural induced EBs. We observed that in both murine and human samples P and S maps from neural induced EBs showed a strong polarization pattern, while P and S maps from spontaneously differentiated EBs showed a diffuse, unpolarized pattern. These facts confirm that polarization of P and S occur concomitant to EB neural differentiation. Further analyses of cell density, proliferation and death also confirmed that neural induced EBs are symmetric in these aspects, an indication that the polarized pattern is not due to areas of high cellular concentration.

There are reports on SR-XRF analyses on neural tissues and neural cells involving pattern alterations of P, S, Cu and Zn on disease or injury. A work by Pushie and colleagues [13] shows that prion protein expression levels alter the metallic elements Fe, Cu and Zn distribution in the mouse brain, these results could be correlated to brain diseases in which metal homeostasis is disrupted, such as Alzheimer's and Parkinson's.

Another work, from Chwiej and colleagues [29] analyzes elemental distribution on rat brains after mechanical injury. In addition to showing that mechanical injury permanently alters atomic distribution, their elemental maps reveal that high concentration areas of P and S overlap, whilst Cu and Zn do not occur in the same areas of P and S in the brain.

P and S are involved in numerous cell processes, their polarization in neural induced EBs indicate additional functions for these elements beyond basal cell maintenance. There are few techniques that enable good P detection and quantification, SR-XRF is well-suited for phosphorus analysis [30].

Also, we found higher levels of total Cu in mES and hES neural stimulated EBs (Figure 5C and 11C, respectively) in comparison to control EBs, a result that can be correlated to the fact that neuronal cells carry large pools of Cu [31]. Cu is a metallic element and is deeply involved in neural tissue activity [32] due to its high ability in transferring single electrons. These data suggest that metal accumulation could be an early event in neural differentiation, triggered in neural progenitor cells.

A work from Wolford and colleagues [33], showed that Zn translocation from the cytoplasm to the nucleus may be an indicator of differentiation and loss of pluripotency in H9 colonies. As SR-XRF analysis has a spatial resolution of a few cells per field, our results show that Zn is indeed spread in the entire EB structure, like in the H9 colonies in Wolford results, although, we cannot confirm if it is cytoplasmic or nuclear Zn. Nevertheless, Zn might have a central role in pluripotent cell dynamics, as its occurrence is preserved from colonies to EBs and from naive to more developed EBs.

Naive EBs showed significant elemental distribution differences between species. 4 days old murine EBs are not naturally polarized, while 7 days old human EBs show polarizations for all four elements, P, S, Cu and Zn. We believe that these differences comprehend intrinsic characteristics of each species. On the other hand, neural stimulated murine and human EBs reach a similar elemental distribution, with P and S polarization. These results reinforce the fact that P and S polarization, in addition to Cu and Zn more spread distribution is linked to neural differentiation on pluripotent stem cells in a broader sense.

How P and S participate in the events of neural differentiation is still unclear, nevertheless their polarizations seem to follow neurogenesis, as high P and S regions give space to high Cu and Zn regions. Cu and Zn do not show strong polarization in any of the conditions; its spread occurrence in different stages of differentiation, along with the fact that metallic elements are widely distributed in neural tissue, reinforces these elements relevancy to stem cell dynamics either in the pluripotent as in the neural differentiated state.

These findings indicate that XRF is a valuable tool to evaluate and compare pluripotent stem cells dynamics, besides being a powerful method to efficiently investigate cellular elements at high sensitivity and resolution ranges. This technique was never before applied on embryoid body analyses, opening a whole new field of possible lines of investigation, highlighting hidden phenomena, such as P and S polarization and the importance of Cu and Zn in *in vitro* neural differentiation.

## Supporting Information

Figure S1 **Embryoid bodies 10 µm cryosections.** Examples of human A) 7 days old; B) 14 days old control; C) 14 days old neural induced embryoid bodies. Examples of mouse R1 D) 4 days old; E) 8 days old control; F) 8 days old neural induced embryoid bodies. Examples of mouse iPS cell G) 4 days old; H) 8 days old control; I) 8 days old neural induced embryoid bodies. Scale bar 100 µm.
(TIF)

Figure S2 **Typical XRF spectra obtained from an irradiated murine embryoid body.** A) Standard spectrum obtained after 100 seconds of exposure. B) Spectrum obtained from the same area as A after 1000 seconds of exposure. Beam area: 20×20 µm.
(TIF)

Figure S3 **Embryoid bodies do not present morphological differences in their original spherical shape.** Bright field images of human embryoid bodies in suspension culture. A) 7 days old; B) 14 days old control non-induced; C) 14 days old neural induced. Scale bar 100 µm for all images.
(TIF)

Figure S4 **Dark areas do not correlate with elemental distribution.** A line) 7 days old human EB without dark areas. B line) Example of other 7 days old human EB that possess a dark area that does not correlate with elemental distribution.
(TIF)

Figure S5 **Murine induced pluripotent stem cells-derived embryoid bodies present similar elemental distribution as mouse embryonic stem cells.** A) Elemental maps; B to E) Measure of elemental content normalized per irradiated area for P, S, Cu and Zn, respectively; F) Symmetry measure: left and right percentage of nuclei for neural induced embryoid bodies; G to I) Left and right intensity of P, S, Cu and Zn in mouse iPS embryoid bodies, 4 days, 8 days control and 8 days neuro, respectively.
(TIF)
