# Supplementary figures and images for: Synchrotron Radiation X-Ray Microfluorescence Reveals Polarized Distribution of Atomic Elements during Differentiation of Pluripotent Stem Cells
Source: PLoS One. 2011 Dec 16;6(12):e29244. doi: 10.1371/journal.pone.0029244 (PMC3241705; doi:10.1371/journal.pone.0029244)

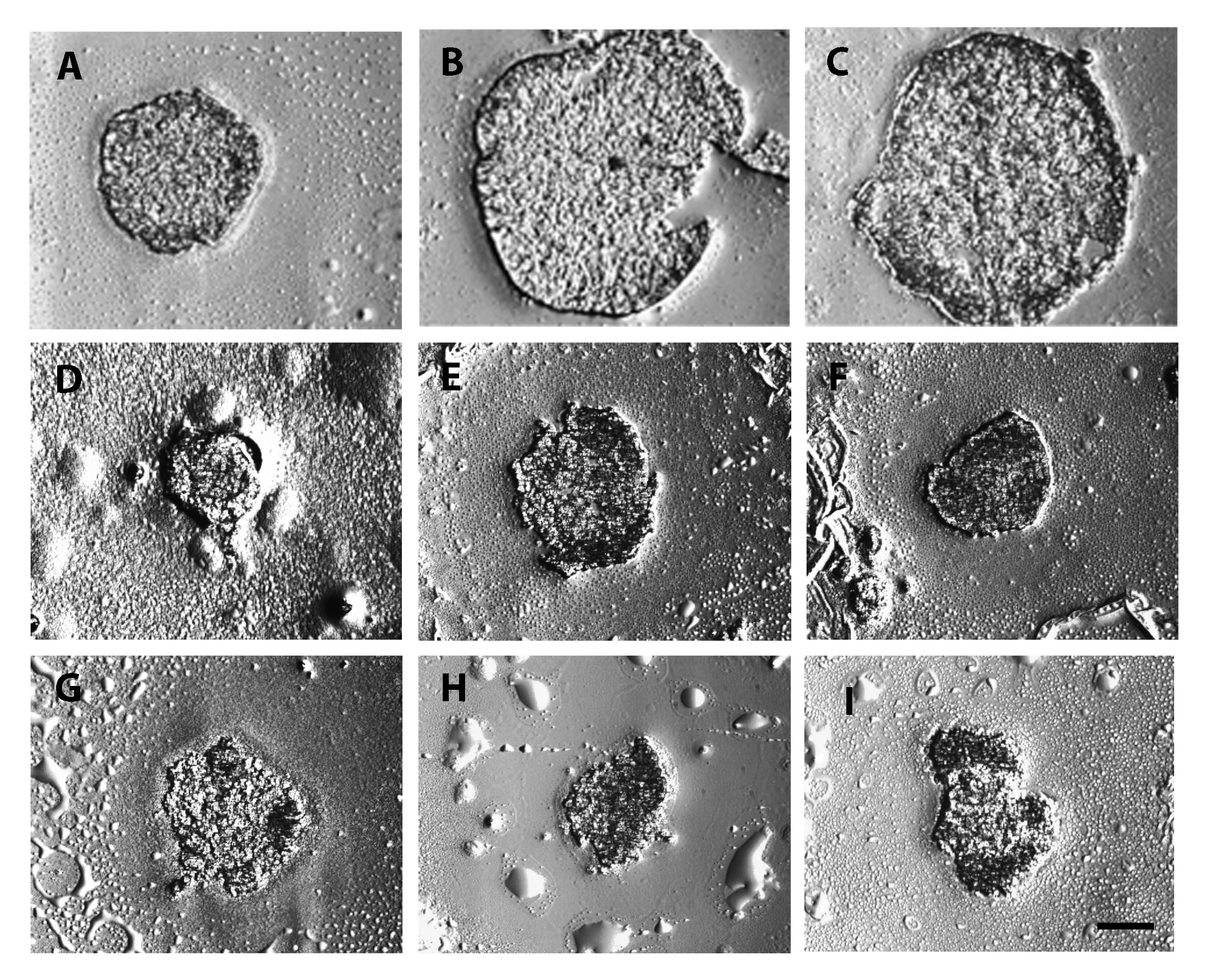

Supplement: Figure S1 — Embryoid bodies 10 µm cryosections. Examples of human A) 7 days old; B) 14 days old control; C) 14 days old neural induced embryoid bodies. Examples of mouse R1 D) 4 days old; E) 8 days old control; F) 8 days old neural induced embryoid bodies. Examples of mouse iPS cell G) 4 days old; H) 8 days old control; I) 8 days old neural induced embryoid bodies. Scale bar 100 µm. (TIF) [file pone.0029244.s001.tif]

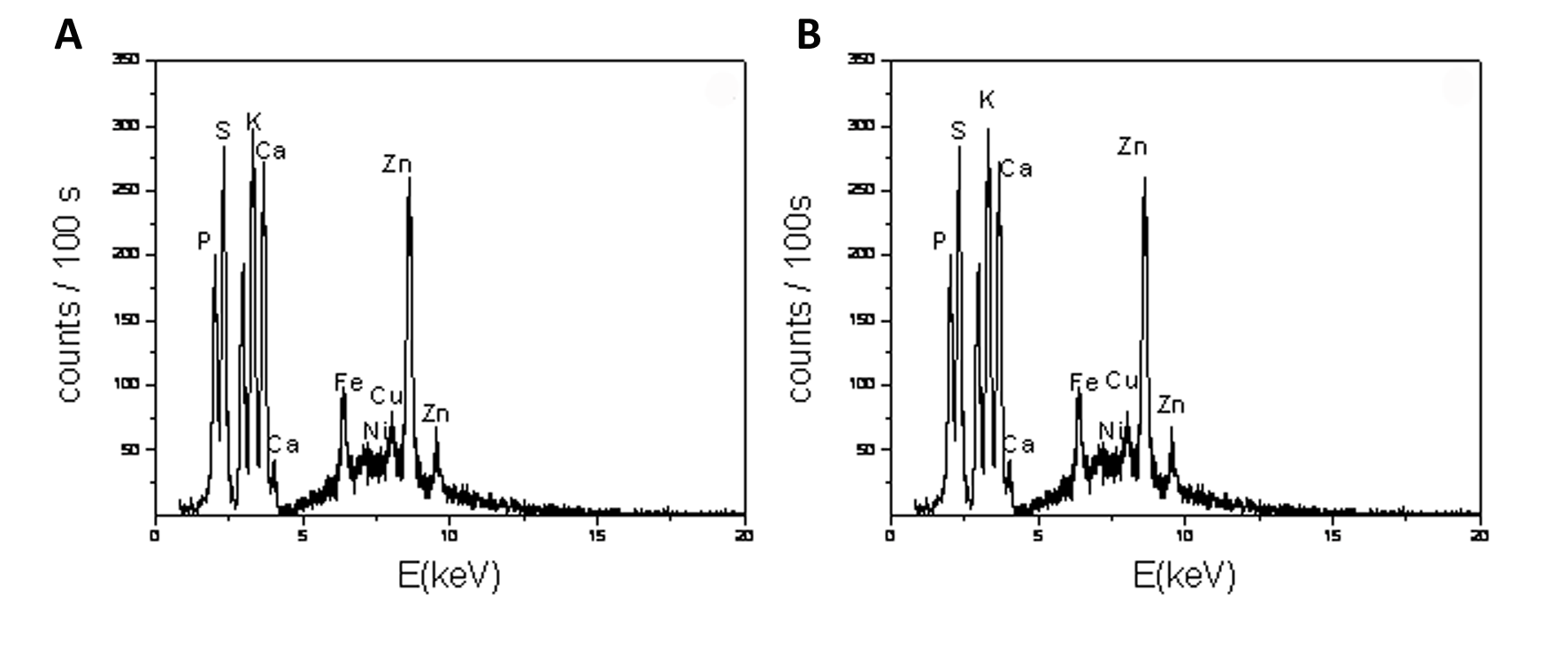

Supplement: Figure S2 — Typical XRF spectra obtained from an irradiated murine embryoid body. A) Standard spectrum obtained after 100 seconds of exposure. B) Spectrum obtained from the same area as A after 1000 seconds of exposure. Beam area: 20×20 µm. (TIF) [file pone.0029244.s002.tif]

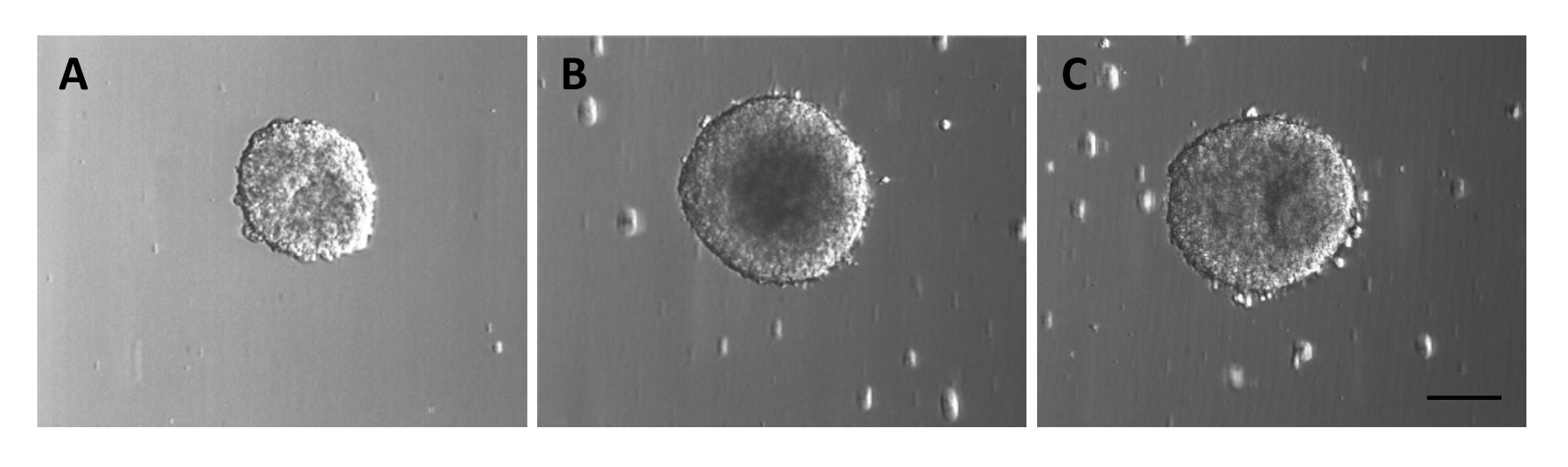

Supplement: Figure S3 — Embryoid bodies do not present morphological differences in their original spherical shape. Bright field images of human embryoid bodies in suspension culture. A) 7 days old; B) 14 days old control non-induced; C) 14 days old neural induced. Scale bar 100 µm for all images. (TIF) [file pone.0029244.s003.tif]

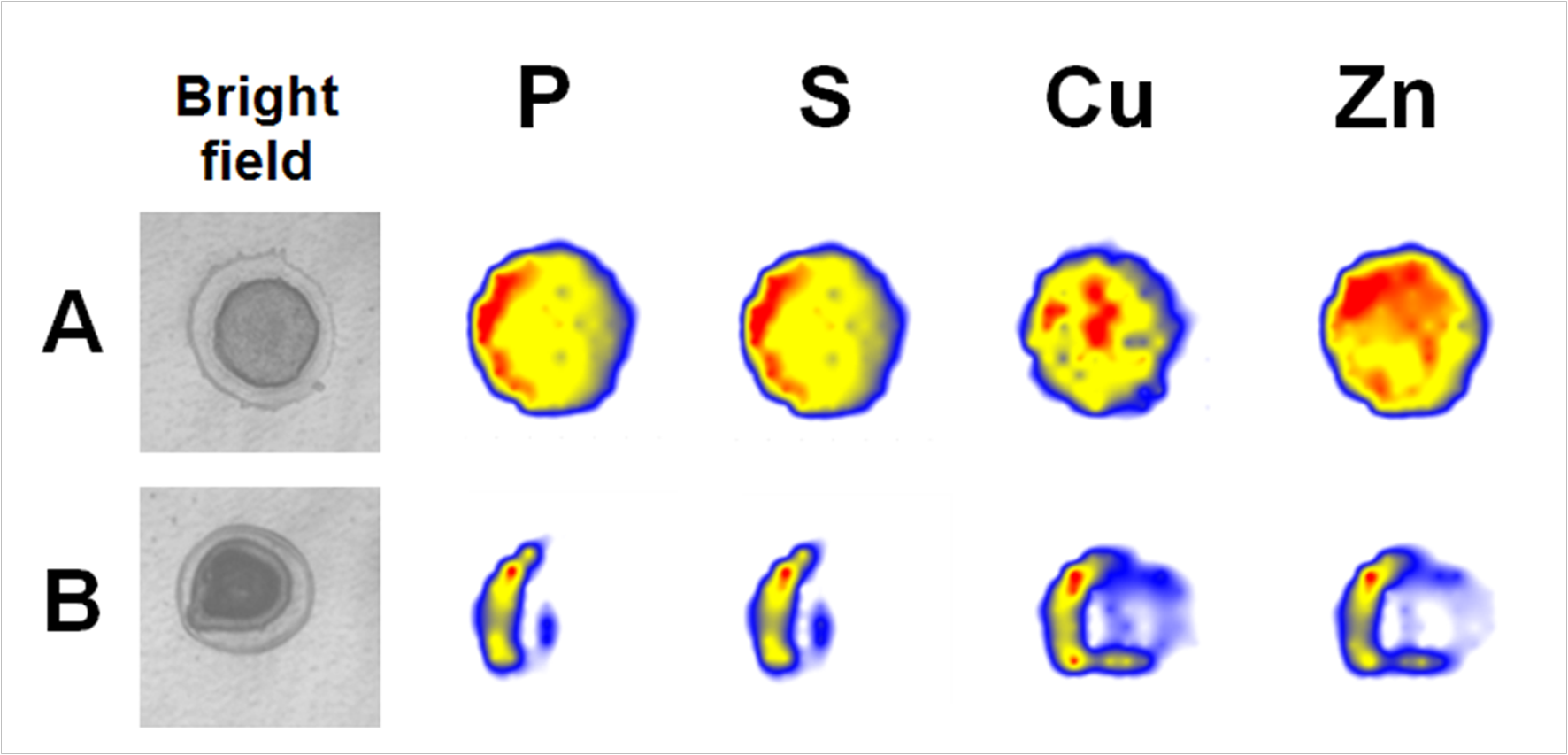

Supplement: Figure S4 — Dark areas do not correlate with elemental distribution. A line) 7 days old human EB without dark areas. B line) Example of other 7 days old human EB that possess a dark area that does not correlate with elemental distribution. (TIF) [file pone.0029244.s004.tif]

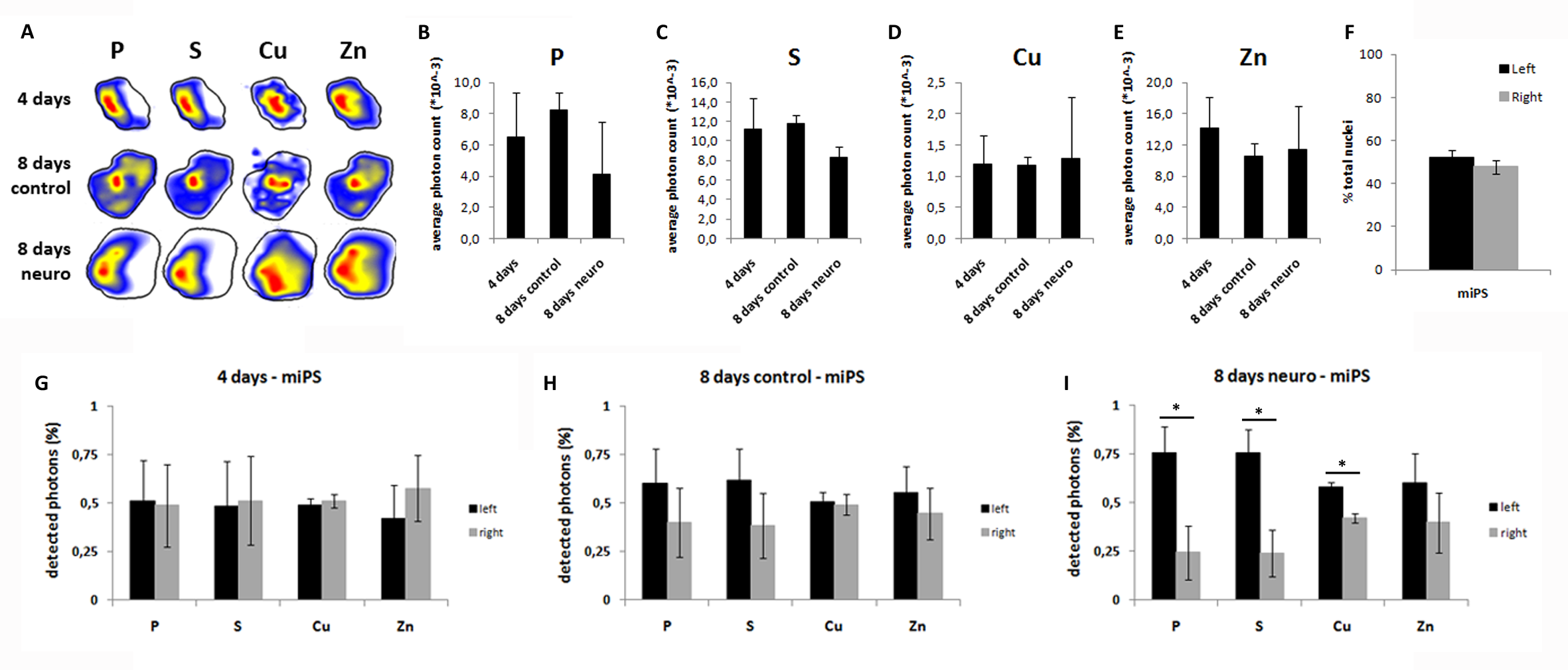

Supplement: Figure S5 — Murine induced pluripotent stem cells-derived embryoid bodies present similar elemental distribution as mouse embryonic stem cells. A) Elemental maps; B to E) Measure of elemental content normalized per irradiated area for P, S, Cu and Zn, respectively; F) Symmetry measure: left and right percentage of nuclei for neural induced embryoid bodies; G to I) Left and right intensity of P, S, Cu and Zn in mouse iPS embryoid bodies, 4 days, 8 days control and 8 days neuro, respectively. (TIF) [file pone.0029244.s005.tif]
